# Cost–utility and cost–benefit analyses of school-based obesity prevention program

**DOI:** 10.1186/s12889-020-09718-x

**Published:** 2020-10-23

**Authors:** Haiquan Xu, Yanping Li, Songming Du, Qian Zhang, Ailing Liu, Junmao Sun, Guansheng Ma

**Affiliations:** 1 Institute of Food and Nutrition Development, Ministry of Agriculture and Rural Affairs, Beijing, China; 2 Department of Nutrition, Harvard T. H. Chan School of Public Health, Boston, MA USA; 3 Chinese Nutrition Society, Beijing, China; 4 National Institute for Nutrition and Health, Chinese Center for Disease Control and Prevention, Beijing, China; 5 Department of Nutrition and Food Hygiene, School of Public Health, Peking University, 38 Xue Yuan Road, Beijing, 100191 China

**Keywords:** Childhood obesity, School-based intervention, Obesity intervention, Cost-utility, Cost-benefit

## Abstract

**Background:**

Economic evaluation of school-based obesity interventions could provide support for public health decision of obesity prevention. This study is to perform cost–utility and cost–benefit assessment of three school-based childhood obesity interventions including nutrition education intervention (NE), physical activity intervention (PA) and comprehensive intervention (both NE and PA, CNP) with secondary data analysis of one randomized controlled trial.

**Methods:**

The standard cost-effectiveness analysis methods were employed from a societal perspective to the health outcome and costs that are attributable to the intervention. NE, PA and CNP were carried out separately for 2 semesters for childhood obesity interventions in primary schools. The additional quality-adjusted life years (QALYs) resulting from the interventions were measured as the health outcome. A cost–utility ratio (CUR) and A cost–benefit ratio (CBR) was calculated as the ratio of implementation costs to the total medical and productivity loss costs averted by the interventions.

**Results:**

The CUR and CBR were ¥11,505.9 ($1646.0) per QALY and ¥1.2 benefit per ¥1 cost respectively, and the net saving was ¥73,659.6 ($10,537.9). The CUR and CBR for nutrition education and physical activity interventions were ¥21,316.4 ($3049.6) per QALY and ¥0.7 benefit per ¥1 cost, ¥28,417.1 ($4065.4) per QALY and ¥0.4 benefit per ¥1 cost, respectively (in 2019 RMB). Compared with PA intervention, the ICERs were ¥10,335.2 ($1478.6) and 4626.3 ($661.8) for CNP and NE respectively. The CBR was ¥1.2, 0.7, and 0.4 benefits per ¥1 cost for CNP, NE, and PA interventions, respectively. Net estimated savings were achieved only through CNP intervention, amounting to ¥73,659.6 ($10,537.9).

**Conclusions:**

Comprehensive school-based obesity intervention is a beneficial investment that is both cost-effective and cost saving. Compared with PA intervention, both CNP and NE intervention were more cost-effective.

**Supplementary information:**

**Supplementary information** accompanies this paper at 10.1186/s12889-020-09718-x.

## Background

The prevalence of obesity has increased rapidly worldwide recently [1–3]. In addition, the epidemic of childhood obesity is becoming one of the most crucial public health challenges globally [4, 5]. Childhood obesity may increase the risk of adult obesity, which likely increases the burdens of several diseases including diabetes, cardiovascular diseases, and other noncommunicable diseases in later life [6]. The increasing prevalence of childhood obesity is also a major economic concern that may produce severe adverse consequences, such as lower skill attainment, poor academic outcomes, reduced labor productivity, and increased health care cost [7].

The prevalence of childhood obesity in China increased nearly 50-fold, from 0.15 to 7.3%, between 1985 and 2014 [8]. To develop the most effective intervention strategy, an increasing number of childhood obesity intervention studies have been implemented in China [9–11]. From a policy development perspective, the cost per quality-adjusted life year (QALY) to the health care system is of particular interest. Because of the wide range of health-related, societal, and economic consequences, policy makers and intervention providers urgently need evidence-based obesity prevention programs. Although systematic reviews of economic evaluations of pediatric health promotion programs have been performed, most of them evaluated the school-based programs in developed countries [12, 13], though some school-based obesity intervention programs have been carried out in China, most of them focused on effects evaluation and rare on economic evaluation. To evaluate the effects and cost-effectiveness of a school-based comprehensive intervention program for childhood obesity, we conducted the nutrition-based comprehensive intervention study on childhood obesity in China (NISCOC).

The NISCOC comprised two trials. One trial was a cluster-randomized controlled trial of separate nutrition education (NE) and physical activity (PA) interventions. In Beijing, eight randomly selected schools were divided into three intervention groups (three schools for NE, three schools for PA, and two schools as shared controls). The other trial was a multicenter cluster-randomized controlled trial of comprehensive interventions including both nutrition education and physical activity (CNP) in five cities: Shanghai, Chongqing, Guangzhou, Jinan, and Harbin. Six schools from each city were randomly assigned to one of two groups (three to CNP and three to control). In total, 38 primary schools were included in this trial. The program was implemented for two semesters from May 2009 to May 2010. NE was targeted toward parents, teachers, and health workers in treatment group schools [14]. Our analysis indicated that CNP could produce a greater preventative effect against childhood obesity than the other two single interventions [15]. The present study performed cost–utility and cost–benefit analyses of the NISCOC childhood obesity interventions.

## Methods

This analysis was conducted from a societal perspective to the health outcome and costs that are attributable to the intervention. The costs of three childhood obesity interventions (CNP intervention, single NE intervention, and single PA intervention) were estimated separately. The QALYs saved from adult (≥ 40 years old) obesity cases prevented were used as the health outcome. The medical and productivity loss costs associated with cases of adulthood obesity that were averted as a result of intervention were estimated. To correspond with the timing of the intervention, all costs were calculated in 2009 RMB, and then translated into 2019 RMB and 2019 US dollars ($1 equals to ¥6.99 in 2019). The imputation began at 10 years old (in 2010). All costs averted and QALYs saved over a 25-year period, 40 to 65 years of age, were calculated and discounted at an annual rate of 3%. Missing data were replaced using imputation methods, some records with missing data of BMI or sex were not included for analysis. This study was approved by the Ethical Review Committee of National Institute for Nutrition and Food Safety. The informed consent voluntarily was signed by participants’ parents or their guardians.

### Intervention implementation

In NE intervention schools, one nutrition education handbook was developed [16] for the students, courses on nutrition and health for the students, parents, teachers and health workers were designed separately. Furthermore, “Dietary Pagoda for Chinese people” posters were displayed in the classrooms. In PA intervention schools, the “Happy 10”, which was a classroom-based physical activity program for primary school students, was used [15, 17]. Furthermore, students, parents, health workers and teachers received the PA education from the program. The parents were also involved for improving the home environment, including but not limited to sending them physical activity education bulletins. In CNP schools, all above interventions including both NE and PA were implemented. The supervisors from project office went to each center to carry out project supervision during the program period.

### Intervention costs

The intervention costs were collected through both accounting records and retrospective methods. Both monetary and labor costs were included in the total intervention costs. Monetary costs comprised the monetary investment during program implementation, and the labor costs refer to transforming labor investment into currency. The intervention costs included different funds and resources spent on project initiation, training, implementation, and supervision. The total costs of CNP, NE and PA were ¥398,851.8 ($57,060.3), ¥63,679.4 ($9110.1) and ¥58,345.1 ($8346.9), respectively (Table 1).

**Table 1:** The intervention costs of three intervention measures in NISCOC (¥ ($))

Item	Resource	CNP	NE	PA
Material and bullet development	Labor	10,579.0 (1513.4)	2023 (289.4)	1840.5 (263.3)
	Money	43,908.4 (6281.6)	8509.8 (1217.4)	5258.0 (752.2)
Training and experience	Labor	1099.6 (157.3)	0	0
	Money	23,622.7 (3379.5)	3714.3 (531.4)	3379.1 (483.4)
Transport fee	Money	27,139.3 (3882.6)	4942.0 (707.0)	4496.0 (643.2)
The other cost for implemention	Labor	247,044.0 (35,342.5)	18,865.1 (2698.9)	20,059.1 (2869.7)
	Money	45,459.0 (6503.4)	25,625.1 (3666)	23,312.5 (3335.1)
Total		398,851.8 (57,060.3)	63,679.4 (9110.1)	58,345.1 (8346.9)

*CNP* comprehensive interventions including both nutrition education and physical activity, *NE* nutrition education, *PA* physical activity, *NISCOC* the nutrition-based comprehensive intervention study on childhood obesity in China

### Cases of adult obesity prevented

The cases of overweight and obesity being prevented in this program was calculated according to the intervention effects on prevalence of overweight and obesity [15]. Height and overnight fasting body weight were measured during physical examination in school at baseline and end. Body mass index (BMI) was calculated as weight in kilograms divided by height in meters squared (kg/m^2^). Childhood overweight was defined as a BMI between the 85th and 95th percentiles, whereas childhood obesity was defined as a BMI in the 95th percentile or above, according to the age- and sex-specific BMI cutoffs developed by the Working Group for Obesity in China [18]. The cases of overweight and obesity prevented by three interventions after this program was calculated respectively as

1 $$ {N}_{2 ij}={N}_t\left(\left({P}_{1b}-{P}_{1e}\right)-\left({P}_{2b}-{P}_{2e}\right)\right), $$

Where

*i* = *m*, *f*, indicating male or female, respectively,

*j* = *ow*, *ob*, representing overweight or obesity, respectively,

*N*_2*ij*_ = Cases of overweight or obesity prevented at the end of this study (at age 10),

*N*_*t*_ = The total participants received the intervention,

*P*_*1b*_ = prevalence of overweight or obesity at baseline in intervention group,

*P*_*1e*_ = prevalence of overweight or obesity after intervention in intervention group,

*P*_*2b*_ = prevalence of overweight or obesity at baseline in control group,

*P*_*2e*_ = prevalence of overweight or obesity after intervention in control group.

The cases of overweight and obesity prevented in the program and after 40 years old were shown in Table 2. The literature data was used to estimate the probability of progressing from overweight or obese child to obese adult. A two-stage overweight/obesity progression model was used to predict adult cases with obesity by age 40. First, the probability at 10 years old of children developing overweight or obesity at ages 21 to 29 was estimated with literature data [19]. Second, the obesity cases averted at ages 40 to 65 were estimated according to the probability of developing obesity at these ages conditional upon obesity at age 21 to 29 [20]. We assumed that adults who developed obesity by age 40 in this model would remain obese until age 65. The obesity progression model was in Appendix.

**Table 2:** The cases of overweight and obesity prevented at the end of the program

	CNP	NE	PA
Cases of overweight or obesity prevented after the program (measured)			
Overweight			
All	83.9	26.4	8.2
Boy	0	22.4	8.2
Girl	83.9	4.0	0
Obesity			
All	46.3	6.7	14.5
Boy	46.3	0	1.4
Girl	0	6.7	13.1
Cases of obesity prevented ≥40 years old (predicted)			
Male	20.2	0.0	3.6
Female	34.3	4.9	0.0

*CNP* comprehensive interventions including both nutrition education and physical activity, *NE* nutrition education, *PA* physical activity

### QALYs saved

The equations for QALY saved estimation and other parameters in our study were drawn from studies by Brown [20], Erickson [21], and Peeters [22]. QALYs saved are the additional QALYs gained by avoiding adult obesity, the data used for the estimate of QALYs saved was from literature. Activity scales were used in QALY estimation to weight and quality-adjust values, with a health status value ranging from 0.10 (limited with poor health) to 1.00 (no limitations with excellent health). The equation used to estimate QALYs saved was in Appendix.

### Productivity loss averted

The averted costs from productivity loss included those costs averted that were associated with losses attributed to impaired work ability for obesity-related morbidity and mortality. The life expectancy, mortality, and additional sick days for 40-year-old individuals with and without obesity who die before 65 were calculated according to literature [20], which used 2002 American National Health Interview Survey data. Wages in the six cities where the program was implemented were used to place value on the productivity lost due to obesity-related illness. The mean annual and daily earnings of full-time workers in the six cities were calculated according to data from the National Bureau of Statistics of China [23]. Equations used to estimate the costs of labor productivity loss per participant were in Appendix.

### Medical costs averted

The prediction model for medical costs averted was developed following Brown and Wang [24]. The data used to estimate the medical costs averted after obesity avoided were drawn from Zhao’s study [25], which calculated the total medical costs from four chronic diseases—hypertension, type 2 diabetes, coronary heart disease, and stroke—attributable to overweight and obesity in China. These costs were estimated using data from the 2002 National Nutrition and Health Survey and 2003 Third National Health Service Survey [26, 27]. The formulas for medical costs averted through adult obesity prevention were in Appendix.

### Cost–utility and cost–benefit analysis

Cost–utility and cost–benefit analyses were applied on the outcomes of the interventions. A cost–utility ratio (CUR), which was defined as the net intervention costs per QALY saved by the intervention, and cost–benefit ratio (CBR), defined as the ratio of intervention costs to the sum of medical and productivity loss costs averted, were used for intervention assessment.

CUR can be written as

2 $$ CUR=\left(C-{N}_{1i}{B}_3\right)/{N}_{1i}Q $$

and the CBR can be written as

3 $$ CBR=C/\left({N}_{1i}B+{N}_{1i}{B}_3\right) $$

where

*C* = cost of intervention,

*N*_*1i*_ = number of cases of adult obesity prevented,

*Q* = QALYs saved per case of obesity prevented,

*B* = productivity loss costs averted per case of obesity prevented,

*B*_3_ = medical costs averted per case of obesity prevented.

The incremental cost-effectiveness ratio (ICER), the difference in cost (USD) divided by the difference in utility (QALYs gained) between the two interventions, was calculated.

### Sensitivity analysis

Sensitivity analysis was conducted to evaluate the extent to which the results were dependent on the intervention effect and cost parameters used and assess the robustness of cost-utility. A Bayesian multivariate probabilistic sensitivity analysis was performed with 10,000 Monte Carlo simulations, using physical activity intervention as the reference arm. For costs, a gamma distribution was used, and for transition probabilities and utilities a beta distribution was used.

## Results

**Fig. 1:**
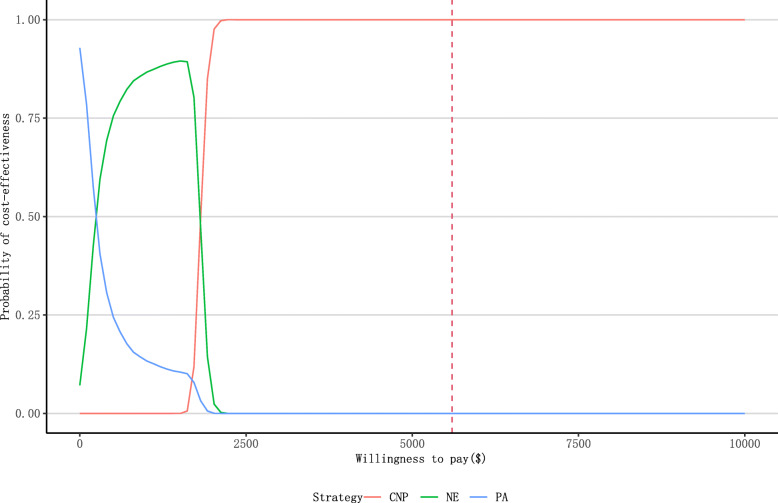
The cost-effective acceptability curve for three interventions

The results of the cost–utility and cost–benefit analyses are displayed in Tables 3 and 4, respectively. The analyses estimated that 30.3 QALYs saved and cost of ¥11,505.9 ($1646.0) per QALY by the CNP intervention, 2.8 QALYs saved and cost of ¥21,316.4 ($3049.6) per QALY by the NE intervention, and 1.9 QALYs saved and cost of ¥28,417.1 ($4065.4) per QALY by the PA intervention. Compared with PA intervention, the ICERs were ¥10,335.2 ($1478.6) and 4626.3 ($661.8) for CNP and NE respectively (Table 3). The CBR was ¥1.2, 0.7, and 0.4 benefits per ¥1 cost for CNP, NE, and PA interventions, respectively. Net estimated savings were achieved only through CNP intervention, amounting to ¥73,659.6 ($10,537.9) (Table 4). Our probabilistic sensitivity analysis revealed that in most of the simulations, the CNP remained cost-effective with 1.0 at a willingness-to-pay threshold of $5000/QALY. (Fig. 1).

**Table 3:** The cost-utility analysis for 3 interventions

	CNP	NE	PA
QALY saved			
Boy	10.8	0.0	1.9
Girl	19.5	2.8	0.0
Total	30.3	2.8	1.9
Intervention cost (C, ¥ ($))	398,851.8 (57,060.3)	63,679.4 (9110.1)	58,345.2 (8346.9)
Medical Care Costs Averted (B_3_, ¥ ($))	50,327.0 (7199.9)	4511.5 (645.4)	3341.0 (478.0)
CUR (¥ ($))	11,505.9 (1646.0)	21,316.4 (3049.6)	28,417.1 (4065.4)
ICER (¥ ($))	10,335.2 (1478.6)	4626.3 (661.8)	–

*CNP* comprehensive interventions including both nutrition education and physical activity, *NE* nutrition education, *PA* physical activity, *C* cost, *CUR* cost–utility ratio, *ICER* incremental cost-effectiveness ratio

**Table 4:** The cost-benefit analysis for 3 different interventions

Intervention type	Intervention costs (¥ ($))	Costs of lost productivity averted (¥ ($))	Medical care costs averted (¥ ($))	Net benefit (¥ ($))	Cost-benefit ratio
CNP	398,851.8 (57,060.3)	422,184.4 (60,398.3)	50,327.0 (7199.9)	73,659.6 (10,537.9)	1:1.2
NE	63,679.4 (9110.1)	42,654.0 (6102.1)	4511.5 (645.4)	−16,513.9(− 2362.5)	1:0.7
PA	58,345.2 (8346.9)	22,028.9 (3151.5)	3341.0 (478.0)	−32,975.3(− 4717.5)	1:0.4

*CNP* comprehensive interventions including both nutrition education and physical activity, *NE* nutrition education, *PA* physical activity

## Discussion

Though two single interventions were negative for the net benefit, all three school-based intervention measures for childhood obesity prevention were cost-effective by cost-utility in this study. Economic analyses revealed that the comprehensive intervention was cost saving, and the cost per QALY saved was less than that of the other two interventions. Effect evaluation of this childhood obesity intervention indicated that comprehensive childhood obesity intervention could significantly control childhood obesity [15]. To perform economic evaluation of obesity interventions, we implemented this study using a life-course approach.

To our knowledge, this is the first study to perform cost–utility and cost–benefit analyses of school-based childhood obesity interventions in China. Although the findings are particularly relevant to public health decision-makers in China, they are also valuable for other countries in similar situations. The comprehensive intervention was net beneficial, whereas the other two were not. We therefore performed the sensitivity analysis on only the comprehensive intervention. Further promotion of the comprehensive intervention could increase the CBR to ¥1.50 benefit per 1 cost. This indicates that comprehensive intervention for preventing childhood obesity could become more beneficial and reduce development costs in further expansion of the program, thus improving the benefit. As noted, few evaluations of childhood obesity interventions include cost–utility and cost–benefit analyses and thus cannot easily be compared with ours; moreover, differences in the age of participants, country, intervention components, and outcome measures make comparison with other studies difficult.

A threshold for cost-effectiveness is generally set to identify sufficiently effective interventions. Although there is no universally accepted standard, several thresholds have been used. Some experts consider health-related programs with CUR < US$30,000 per QALY saved are generally cost-effective [28–30]. The most commonly used cost-effectiveness thresholds are those based on a country’s per capita gross domestic product (GDP) [31]. According to the World Health Organization, interventions with an incremental cost-effectiveness ratio (ICER) per QALY less than one GDP per capita can be considered highly cost-effective. Those with an ICER 1–3 times GDP per capita can be considered cost-effective, and those with an ICER > 3 times the GDP per capita are not considered cost-effective [32]. China’s GDP per capita was ¥29,992(2019 $5599.4) in 2010 [33]. According to the aforementioned standards, the comprehensive intervention and two single interventions were cost-effective, and the comprehensive intervention was the most cost-effective. Compared with physical activity intervention, the ICERs for comprehensive intervention (US$1478.6/QALY) and nutrition education (US$661.8/QALY) were both lower than one GDP. The Planet Health program was a 2-year school-based intervention designed to reduce obesity rates among middle school students in the United States, but the prevalence of obesity only among girls was reduced significantly. The cost was US$4305 per QALY saved and the net savings was US$7313 to society among girls [24]. Although all scenarios were cost-effective and most were cost saving, compared with our results, the cost per QALY saved was greater. One randomized controlled trial enrolling 80 participants (aged 8–19 years) with severe obesity was implemented to conduct an economic evaluation in the Netherlands. Participants received an intensive 1-year lifestyle program with an inpatient period of either 2 months (short-stay group) or 6 months (long-stay group). The program focused on nutrition, PA, and behavior change and required active participation from parents and caregivers. Although the short-stay treatment was considered more cost-effective, the results indicated no difference in QALYs saved between the groups after 24 months [34]. The coordinated approach to child health (CATCH) intervention program promoted healthy eating and PA in US elementary schools between 2000 and 2002. CATCH was cost-effective and net beneficial, costing $900 per QALY saved, and the net benefit was $68,125 (2004 US dollars) [20], which was more cost-effective than our intervention. Generally, childhood obesity prevention is considered more cost-effective than adult obesity treating, and our study provides evidence to support this. Treating adult obesity with the drugs orlistat or sibutramine or through gastric bypass surgery costs US$8327, US$9299, and US$5000–$35,600 per QALY, respectively, and Wheeling Walks, a behavior modification program for sedentary adults, costs $14,286 per QALY [35]. These treatments were much more costly than the comprehensive childhood intervention evaluated here.

Forecasting methods were used to predict the long-term obesity prevention effect [19–22, 24]. By taking into account the natural risk of obesity in general cohort, it could exclude the probability of obesity under natural transition to predict the obesity processing rate in adulthood. In addition, the predictions of QALYs saved, medical cost averted and productivity losses averted were from the life perspective, which made the prediction results closer to the real situation. This study has some limitations to acknowledge. First, many factors of this cost-effectiveness analysis are unknown, such as how effectively participants-maintained weight loss over time. Second, some parameters used in QALY estimation were drawn from other countries. These countries may differ from China; however, no referred studies of a Chinese cohort were available. Third, the benefits evaluated were not comprehensive. The health benefits that may arise from improvements in health knowledge, attitudes, self-efficacy, and leadership of parents, teachers, and school canteen staff were not included. Therefore, our CUR per QALY gained estimates may be conservative.

## Conclusions

According to our model analysis, school-based comprehensive childhood obesity intervention is cost-effective for obesity prevention and reduction of lifetime chronic disease risk. Such school-based comprehensive programs are likely to reduce the public health care burden from obesity and chronic diseases.

## Supplementary information

**Additional files 1.** Appendix.

## Data Availability

The datasets enabling this research are not publicly available due to privacy or ethical restrictions but are available from the corresponding author on reasonable request.

## Abbreviations

QALY: Quality-adjusted life year
CUR: Cost–utility ratio
CBR: Cost–benefit ratio
NISCOC: The nutrition-based comprehensive intervention study on childhood obesity in China
NE: Nutrition education
PA: Physical activity
CNP: Comprehensive interventions including both nutrition education and physical activity
BMI: Body mass index
CIs: Confidence intervals
GDP: Gross domestic product
ICER: Incremental cost-effectiveness ratio
CATCH: The coordinated approach to child health
