# Supplementary material for: Cost–utility and cost–benefit analyses of school-based obesity prevention program
Source: BMC Public Health. 2020 Oct 23;20:1608. doi: 10.1186/s12889-020-09718-x (PMC7585177; doi:10.1186/s12889-020-09718-x)
Supplement: Supplementary file 1 — Additional files 1. Appendix. [file 12889_2020_9718_MOESM1_ESM.docx]

**Appendix**

The obesity progression model is

$N_{1i}=\left( P_{5i}-P_{6i} \right)\sum_{j} {(N}_{2ij}\times(P_{3ij}-P_{4ij}))$, (1)

where

*i* = *m*, *f*, indicating male or female, respectively,

*j* = *ow*, *ob*, representing overweight or obesity, respectively,

*N*_1_*_i_* = Cases of obesity after age 40 prevented,

*N*_2_*_ij_* = Cases of overweight or obesity prevented at the end of this study (at age 10),

*P*_3_*_ij_* = probability of developing obesity at age 21 to 29 conditional on having obesity (overweight) at age 10,

*P*_4_*_ij_* = probability of developing obesity at age 21 to 29 conditional on not having obesity (overweight) at age 10,

*P*_5_*_i_* = probability of developing obesity at age 40 to 65 conditional on having obesity at age 21 to 29,

*P*_6_*_i_* = probability of developing obesity at age 40 to 65 conditional on not having obesity at age 21 to 29 (Table S1).

| **Table S1** Parameters for predicting adulthood obesity | | | | | |
| --- | --- | --- | --- | --- | --- |
|  |  |  | *i* | |  |
| Parameters | Group | *j* | *m* | *f* | Sources |
| *N_2ij_* | CNP | *ov* | 0 | 83.9 | NISCOC |
|  | CNP | *ob* | 46.3 | 0 | NISCOC |
|  | NE | *ov* | 22.4 | 4 | NISCOC |
|  | NE | *ob* | 0 | 6.7 | NISCOC |
|  | PA | *ov* | 8.2 | 0 | NISCOC |
|  | PA | *ob* | 1.4 | 13.1 | NISCOC |
| *P_3ij_* | *All* | *ov* | 0.69 | 0.69 | [Ref.1] |
|  | *All* | *ob* | 0.83 | 0.83 | [Ref.1] |
| *P_4ij_* | *All* | *ov* | 0.13 | 0.13 | [Ref.1] |
|  | *All* | *ob* | 0.16 | 0.16 | [Ref.1] |
| *P_5i_* | *All* | - | 0.77 | 0.85 | [Ref.2] |
| *P_6i_* | *All* | - | 0.12 | 0.12 | [Ref.2] |

NISCOC: The nutrition-based comprehensive intervention study on childhood obesity in China. CNP, comprehensive interventions including both nutrition education and physical activity. NE, nutrition education. PA, physical activity.

The equation used to estimate QALYs was

$Q=\frac{M_{n}S_{n}\left[ \frac{1}{r}-\frac{1}{r\left( 1+r \right)^{\text{Ln}}} \right]-M_{o}S_{o}\left[ \frac{1}{r}-\frac{1}{r\left( 1+r \right)^{\text{Lo}}} \right]+\left[ \left( 1-M_{n} \right)S_{n}-\left( 1-M_{o} \right)S_{o} \right]\left[ \frac{1}{r}-\frac{1}{r\left( 1+r \right)^{\text{25}}} \right]}{\left( 1+r \right)^{p}}$, (2)

where

*Q* = QALYs saved per participant through obesity prevention,

*S_n_* = activity scale score of a participant without obesity, by sex,

*S_o_* = activity scale score of a participant with obesity, by sex,

*M_n_* = probability of an adult without obesity dying between age 40 and 64, by sex,

*M_o_* = probability of an adult with obesity dying between age 40 and 64, by sex,

*L_n_* = life expectancy of an adult without obesity at age 40 who dies by age 65, by sex,

*L_o_* = life expectancy of an adult with obesity at age 40 who dies by age 65, by sex, and

*r* = annual discount rate (Table S2).

Equations used to estimate the costs of labor productivity loss per participant were written as

$B=B_{1}+B_{2}$, (3)

where

$B_{1}=\frac{W_{d}\left\{ M_{o}D_{o}\left[ \frac{1}{r}-\frac{1}{r\left( 1+r \right)^{\text{Lo}}} \right]-M_{n}D_{n}\left[ \frac{1}{r}-\frac{1}{r\left( 1+r \right)^{\text{Ln}}} \right]+\left[ \left( 1-M_{o} \right)D_{o}-\left( 1-M_{n} \right)D_{n} \right]\left[ \frac{1}{r}-\frac{1}{r\left( 1+r \right)^{\text{25}}} \right] \right\}}{\left( 1+r \right)^{30}}$ (4)

and

$B_{2}=\frac{W_{y}\left\{ M_{n}\left[ \frac{1}{r}-\frac{1}{r\left( 1+r \right)^{\text{Ln}}} \right]-M_{o}\left[ \frac{1}{r}-\frac{1}{r\left( 1+r \right)^{\text{Lo}}} \right]+\left( M_{o}-M_{n} \right)\left[ \frac{1}{r}-\frac{1}{r\left( 1+r \right)^{\text{25}}} \right] \right\}}{\left( 1+r \right)^{30}}$, (5)

where

*B* = averted costs from lost productivity per case of adult obesity prevented,

*B*_1_ = averted costs from lost productivity due to morbidity per case of adult obesity prevented,

*B*_2_ = averted costs from lost productivity due to mortality per case of adult obesity prevented,

*D_n_* = annual mean workdays lost for adults without obesity by sex,

*D_o_* = annual mean workdays lost for adults with obesity by sex,

*W_d_* = mean daily earnings,

*W_y_* = mean annual earnings, and

*r* = annual discount rate (Table S2).

The medical costs averted through adult obesity prevention were calculated with the formulas

$B_{3}=\frac{C_{mca}\left\{ M_{o}\left[ \frac{1}{r}-\frac{1}{r\left( 1+r \right)^{\text{Lo}}} \right]+\left( 1-M_{o} \right)\left[ \frac{1}{r}-\frac{1}{r\left( 1+r \right)^{\text{25}}} \right] \right\}}{\left( 1+r \right)^{\text{30}}}$ (6)

and

$C_{mca}=\frac{C_{tmc}}{P\times N_{a}}\times\left( 1+r \right)^{y}$, (7)

where

$B_{3}$ = lifetime medical costs averted through obesity prevention per participant,

$C_{mca}$= annual medical costs for adult obesity per participant,

$C_{tmc}$ = total medical cost for the four chronic diseases attributable to overweight and obesity in 2003,

*P* = prevalence of adult overweight and obesity in 2002 in China,

*N_a_ =* adult population in 2000 in China,

*r* = annual discount rate, and

*y* = years from the year medical costs were calculated (2002) to the end of the intervention (2009) (Table S2).

| **Table S2** Parameters for predicting adulthood QALYs | | | | | |
| --- | --- | --- | --- | --- | --- |
|  |  |  | *i* | |  |
| Parameters | Group | *j* | *m* | *f* | Sources |
| *M_n_* | *All* | - | 0.079 | 0.066 | [Ref.3] |
| *M_o_* | *All* | - | 0.147 | 0.145 | [Ref.3] |
| *L_n_* | *All* | - | 18.57 | 16.94 | [Ref.3] |
| *L_o_* | *All* | - | 18.46 | 16.8 | [Ref.3] |
| *D_n_* | *All* | - | 1.33 | 1.46 | [Ref.2] |
| *Do* | *All* | - | 1.88 | 2.02 | [Ref.2] |
| *W_d_* | *All* | - | 157.2 | 157.2 | [Ref.4] |
| *W_y_* | *All* | - | 40872 | 40872 | [Ref.4] |
| *C_mca_* | *All* | - | 99.1 | 99.1 | [Ref.4, 5] |
| *S_n_* | *All* | - | 0.872 | 0.859 | [Ref.2] |
| *S_o_* | *All* | - | 0.807 | 0.795 | [Ref.2] |
| *P* | *All* | - | 29.9 | 29.9 | [Ref.6] |
| *N_a_* | *All* | - | 0.876 *billion* |  | [Ref.7] |
| *r* | *All* | - | 0.03 | 0.03 |  |

Reference

Ref.1 Whitaker RC, Wright JA, Pepe MS, Seidel KD, Dietz WH. Predicting obesity in young adulthood from childhood and parental obesity. New Engl J Med. 1997;337: 869-73.

Ref.2 Brown HS 3rd, Pérez A, Li YP, Hoelscher DM, Kelder SH, Rivera R. The cost-effectiveness of a school-based overweight program. Int J Behav Nutr Phys Activ, 2007;4:47. doi:10.1186/1479-5868-4-47.

Ref.3 Peeters A, Barendregt JJ, Willekens F, Mackenbach JP, Mamun AA, Bonneux L, et al. Obesity in adulthood and its consequences for life expectancy: a life-table analysis. Annals of Internal Medicine. 2003;138:24-31.

Ref. 4 National Bureau of statistics. China statistical yearbook 2009. http://www.stats. gov.cn/tjsj/ndsj/2009/ indexch.htm. Accessed 26 July 2019.

Ref.5 Zhao W, Zhai Y, Hu J, Wang J, Yang Z, Kong L, et al. Economic burden of obesity related chronic disease in mainland China. Obes Rev. 2008;9(Suppl 1):62-

Ref.6 Wang L. Report of China Nationwide Nutrition and Health Survey 2002 (1): Summary Report. People’s Medical Publishing House: Beijing, 2005.

Ref.7 National Bureau of statistics. 2000 Chinese people census data. http://www.stats.gov.cn/tjsj/ndsj/renkoupucha/ 2000pucha/pucha.htm.
